# Prescription of oral short-acting beta 2-agonist for asthma in non-resource poor settings: A national study in Malaysia

**DOI:** 10.1371/journal.pone.0180443

**Published:** 2017-06-29

**Authors:** May Chien Chin, Sheamini Sivasampu, Ee Ming Khoo

**Affiliations:** 1 Healthcare Statistics Unit, National Clinical Research Centre, Kuala Lumpur, Malaysia; 2 Department of Primary Care Medicine, Faculty of Medicine, University of Malaya, Kuala Lumpur, Malaysia; Universite de Bretagne Occidentale, FRANCE

## Abstract

**Objective:**

Use of oral short-acting beta 2-agonist (SABA) persists in non-resource poor countries despite concerns for its lower efficacy and safety. Utilisation and reasons for such use is needed to support the effort to discourage the use of oral SABA in asthma. This study examined the frequency of oral short-acting Beta 2-agonist (SABA) usage in the management of asthma in primary care and determined correlates of its usage.

**Methods:**

Data used were from the 2014 National Medical Care Survey in Malaysia, a nationally representative survey of primary care encounters (weighted n = 325818). Using methods of analysis of data for complex surveys, we determined the frequency of asthma diagnosis in primary care and the rate of asthma medication prescription, which includes oral SABA. Multivariate logistic regression models were built to assess associations with the prescription of oral SABA.

**Results:**

A weighted estimate of 9241 encounters presented to primary care with asthma in 2014. The mean age of the patients was 39.1 years. The rate of oral SABA, oral steroids, inhaled SABA and inhaled corticosteroids prescriptions were 33, 33, 50 and 23 per 100 asthma encounters, respectively. It was most commonly used in patients with the age ranged between 20 to less than 40 years. Logistic regression models showed that there was a higher odds of oral SABA usage in the presence of respiratory infection, prescription of oral corticosteroids and in the private sector.

**Conclusion:**

Oral SABA use in asthma is found to be common in a non- resource poor setting and its use could be attributed to a preference for oral medicines along undesirable clinical practices within a fragmented health system.

## Introduction

Asthma affects an estimated 300 million people worldwide [1] and was ranked the 15^th^ most important disorder in the world in terms of extent and duration of disability [2]. Effective disease management remains pivotal for disease control. Principle management of asthma is well established and numerous clinical practice guidelines recommend the use of inhaled short-acting beta-agonist (SABA) as a reliever for acute asthma symptoms and early initiation of low dose inhaled corticosteroid (ICS) [1, 3–6]. The usage of oral SABA, on the other hand, has been discouraged by various asthma guidelines as a result of its safety and efficacy concerns [1, 3–6]. Numerous clinical trials have shown that oral SABA requires higher doses to produce similar efficacy as the inhaled form, leading to more adverse effects such as tachycardia, hyperactivity, decreased oxygen saturation and tremors [7–9].

In a review for possible deletion of oral SABA from the World Health Organisation (WHO) Model of Essential Medicines List (EML) in 2010, it was concluded that the oral dosage forms would only be considered in the absence of the inhaled alternatives as a result of affordability issue of salbutamol inhalers [10, 11]. However, we are still observing widespread and regular use of oral SABA even in non-resource poor countries, including Australia, Singapore, Hong Kong, the United States and Malaysia [12–16]. A study on asthma prescribing preferences among 226 clinicians in Malaysia in 2005, found that over 50% of doctors would prescribe oral SABA as the first line of treatment; the top reasons given were cost and patients’ reluctance to use inhaled salbutamol [16].

The continual use of oral SABA in asthma in non-resource poor countries including Malaysia prompts investigations into the reasons of such usage. As such, this study aims to examine the extent of current prescription of oral SABA in the management of asthma in primary care, and correlates of its usage.

## Methods

### Settings

Health care resources are defined as means in a health system to deliver health care services to the population. It can be grouped into three categories: infrastructure, materials or consumables and human resources. A setting is considered resource adequate if the level of resources meet the global norms for resources in a functioning health system [17]. Malaysia is a country with a dual health system that consists of the public and private sector. It meets the demand for consumable resources in asthma treatment as the country records availability of three essential inhaled medicines (beclomethasone, budesonide and salbutamol) as listed in the WHO EML in both sectors. These medicines are available and found to be affordable, where a generic inhaler of budesonide or salbutamol cost less than one day of wages per inhaler in private pharmacies while an innovator inhaler of beclomethasone cost almost three days wages per inhaler. On the other hand, all medicines prescribed in the public sector are provided free to the patients [18].

### Data source and patient identification

We used data from the Malaysia 2014 National Medical Care Survey (NMCS). This was a national survey of doctor-patient encounters in both public and private primary care that aimed to provide information on the utilization, morbidity pattern and the process of care of primary care visits. Details of the study methodology have previously been published and are described briefly here [19].

The 2014 NMCS utilised a stratified, four-stage sampling design. Firstly, primary care clinics were stratified by states and by sectors (public and private). The first stage of sampling involved random selection of clinics (primary sampling units) based on random numbers generated using Microsoft Excel 2007; the second stage involved random selection of a survey date; the third stage involved sampling of all providers providing care on each date; and the fourth stage involved non-probability sampling of encounters from each clinic on the specified date.

As a provider-based survey, the health care providers were asked to record information describing the patient demographics from each patient encounter as well as clinical information, including problems managed and medications prescribed. The International Classification of Primary Care Second Edition (ICPC-2 Plus) was used to classify the clinical information while the medications were classified using the Anatomical Therapeutic Chemical (ATC) classification [20, 21].

In this study, primary care patient visits with physician-diagnosed asthma (R96-, ICPC-2 code) were evaluated. Data used for this study included age, gender, ethnicity, types of income, presence of asthma exacerbation, presence of respiratory tract infection, prescriptions of oral SABA, oral corticosteroids, ICS, inhaled SABA, antibiotics, and sector.

Survey weights were applied to obtain unbiased estimates of features describing the population from which the samples were drawn, accounting for the sampling stages, clinic non- response and activity weight. An activity weight of a clinic is calculated by the average patient encounters of the clinic per day divided by the number of patient encounters surveyed from the clinic. The complex multistage sampling features of NMCS were taken into account for the effects of stratification and clustering on variance estimates and the Taylor series linearization method for variance estimation was utilised. The prescription data for asthma were first categorised into 11 categories: inhaled SABA; inhaled corticosteroids (ICS); inhaled anticholinergic; oral corticosteroids (oral CS); oral SABA; oral xanthine; leukotriene receptor antagonist (LTRA); combined inhaler; unspecified inhaler; antibiotics; and non-asthma medication (other non-recommended medication prescriptions for asthma treatment). Then, we determined the utilization of medications by dividing the estimated number of medications prescribed for each drug category by the estimated total number of visits for asthma; the results were reported in number of prescriptions per 100 encounters.

Ethics approval for the study to be conducted has been obtained from the Medical Research and Ethics Committee of the Ministry of Health Malaysia (NMRR-09-842-4718).Verbal consent from a health provider representative of each clinic was obtained. A public notice was placed at each participating clinic to inform patients of the ongoing study and to clarify that data would be collected for research purposes only. Patients who did not wish to participate in the study would inform the doctors to opt out from the study.

### Statistical analyses

The outcome of interest was the receipt of oral SABA (R03CC, ATC code). The independent variables included in the analyses were age, gender, ethnic group, types of income, presence of asthma exacerbation (yes:R96002, R96003, R96005, R96007, R96008, R96010; no: R96001, ICPC-2 Plus code), oral corticosteroids co-prescription (H02, ATC code), inhaled corticosteroids (R03BA, ATC code), inhaled SABA co-prescription (R03AC, ATC code), antibiotics co-prescription (J01, ATC code), concomitant respiratory tract infection (R74-R81 and R83, ICPC-2 Plus code) and sector (public/private).

A logistic regression model was performed to determine the associated factors of oral SABA prescription; variables included in the final model were determined by a purposeful selection technique proposed by Hosmer and Lemeshow [22, 23]. Design-adjusted Wald test was used to determine the variables for the multivariate analysis. Variables with P-value of less than 0.25 were selected for the multivariate model. The use of a higher significance level in the initial variable selection is to identify and review important variables before a decision is reached for the final model [23]. In the iterative process of variable selection, covariates which were not significant (at alpha level 0.1) and not confounding (change of greater than 15 to 20% of any remaining parameter estimates compared to the full model) were excluded from the model. Interactions among variables in the model were examined and their significance was determined using the design-adjusted Wald test. We considered all possible interactions between two variables in the model. The significance level used was 0.05. Subsequently, the preliminary final model was assessed for its fit using the Hosmer-Lemeshow design-adjusted test [24].

The analyses were performed using multiple imputed data sets to account for item- missing data. The sequential regression approach with application of the Monte Carlo Markov Chain Gibbs sampler algorithm was used [25]. The imputation model was specified by inclusion of all analysis variables, variables that are associated with the analysis variables and variables identifying the complex design features. By incorporating the survey weights and sampling error codes, the imputation model takes into account the possible associations between design variables and the survey variable of interest to reduce bias in the multiple imputation estimates [26]. All analyses were conducted using Stata version 14.1(STATA Corp., TX, USA) [27].

## Results

### Descriptive analyses

The 2014 NMCS data represented 325818 patient encounters at primary care. A weighted estimate of 9241 encounters presented to primary care with asthma in 2014; the rate of visit for asthma was about 3 per 100 patient encounters. The baseline characteristics of these encounters are shown in Table 1. The estimated mean age of the patients with asthma encounters was 39.1 years, 52.4% were male, and the majority were of Malay ethnicity (79.0%). Almost half of the asthma encounters were patients who had own income (54.1%) and 54.5% were seen in the private sector. About 23.6% and 24.4% of the asthma encounters had presence of respiratory infection and asthma exacerbations respectively.

**Table 1:** Characteristics of asthma encounters (N = 9241).

Category	Weighted % of asthma encounters (weighted counts)	95% CI of percentages of asthma encounters
**Age**		
<1	1.4(127)	0.3–3.4
1 to <5	5.7(525)	3.4–7.9
5 to <20	13.6(1254)	10.0–17.1
20 to <40	28.8(2663)	23.6–34.0
40 to <60	32.6(3014)	27.8–37.4
≥60	18.0(1659)	13.2–22.7
**Gender**		
Male	52.4(4840)	47.1–57.7
Female	47.6(4400)	42.3–52.9
**Ethnicity**		
Malay	79.0(7302)	74.1–84.0
Chinese	6.9(636)	4.1–9.7
Indian	8.3(771)	5.4–11.3
Others	5.8(531)	3.1–8.4
**Income type**		
No income	21.8(2014)	17.8–25.8
Own Income	54.1(5000)	47.6–60.6
Parental Income	18.1(1677)	12.9–23.4
Pension	7.0(550)	1.4–10.6
**Asthma exacerbation**		
No	75.6(6982)	70.0–81.1
Yes	24.4(2259)	18.9–30.0
**Respiratory infection**		
No	76.4(7058)	71.1–81.7
Yes	23.6(2182)	18.3–29.0
**Sector**		
Public	45.5(4206)	35.6–55.5
Private	54.5(5435)	44.5–64.4

CI- confidence interval

Table 2 shows the number of visits per 100 encounters where each medication category was prescribed. The most common prescribed drugs in descending order were non-asthma drugs (60 per 100 encounters), inhaled SABA (50 per 100 encounters), oral corticosteroids (33 per 100 encounters) and oral SABA (33 per 100 encounters).

**Table 2:** Frequency of asthma prescriptions.

Medication category (%)	Frequency	Rate per 100 encounters	95% CI
Non-asthma medications	5555	60.1	51.0–69.2
Inhaled SABA	4639	50.2	43.1–57.3
Oral CS	3034	32.8	24.6–41.1
Oral SABA	3013	32.6	24.7–40.6
Inhaled CS	2160	23.4	16.7–30.0
Antibiotics	1723	18.7	12.4–24.9
Xanthine	1653	17.9	9.9–25.8
Combination inhaler	1380	14.9	9.3–20.5
Inhaled, unspecified	400	4.3	2.0–6.6
LTRA	267	2.9	1.4–4.4
Inhaled anticholinergic	246	2.7	1.3–4.0

CI- confidence interval

### Logistic regression models of prescription of oral SABA

Tables 3 and 4 show the results of the bivariate analyses and two final logistic regression models for associations of prescription of oral SABA. The final models included the following predictor variables: asthma exacerbation, oral corticosteroids prescription, ICS, inhaled SABA prescription, concomitant respiratory infection and sector. Significant interactions between prescription of inhaled SABA versus prescription of oral corticosteroids and inhaled SABA versus sector were identified and included in the final models. To consider all possible interactions between two variables, we presented final model 1 with ‘not given inhaled SABA’ as the reference group and final model 2 with ‘given inhaled SABA’ as the reference group in Table 4. The Hosmer-Lemeshow tests across the original and imputed data sets showed an overall good fit of the model (F(9,211–220): 0.6–1.0, p value ranges from 0.4 to 0.7).

**Table 3:** Binary logistic regression models of factors associated with oral SABA prescription weighted multiple imputation estimates.

Parameter	Category	%	OR (95% CI)	F test
Age	<1	1.7	—	F(6,515) = 3.2 p<0.25
	1-<5	8.5	1.4(0.33–6.20)	
	5-<20	18.1	1.2(0.3–5.2)	
	20-<40	34.2	1.0(0.2–4.6)	
	40-<60	29.3	0.6(0.1–2.90)	
	> = 60	8.3	0.3(0.1–13)	
Gender	Female	47.5	—	F(1,510.5) = 0 p = 1
	Male	52.5	1.0(0.6–1.6)	
Ethnicity	Malay	76.3	—	F(3,287.1) = 0.7 p = 0.6
	Chinese	8.6	1.4(0.6–3.0)	
	Indian	8.0	1(0.5–2.2)	
	Others	7.1	1.8(0.7–4.5)	
Income type	No income	15.6	—	F(3,327) = 3.45 p<0.25
	Own Income	56.3	1.6(1.0–2.7)	
	Parental Income	25.3	2.9(1.5–5.9)	
	Pension	2.7	0.6(0.2–2.0)	
Asthma exacerbation	No	59.0	—	F(1,515) = 19.6***
	Yes	41.0	3.5(2.0–6.2)	
Oral corticosteroids	Not given	43.8	—	F(1,515) = 32.2***
	Given	56.2	4.7(2.7–8.0)	
Inhaled corticosteroids	Not given	0.9	—	F(1,515) = 19.1***
	Given	0.1	0.09(0.0–0.3)	
Inhaled SABA	Not given	73.7	—	F(1,515) = 19.9***
	Given	26.3	0.2(0.1–0.4)	
Antibiotics	Not Given	67.2	—	F(1,515) = 9.9 p<0.25
	Given	32.8	3.6(1.6–8.1)	
Respiratory tract infection	No	63.2	—	F(1,515) = 12.5***
	Yes	36.8	2.8(1.6–4.9)	
Sector	Public	15.8	—	F(1,515) = 42.3***
	Private	84.2	8.0(4.3–14.9)	

Results based on multiple imputed datasets

***p<0.001 based on logistic regression results

OR- odds ratio; CI- confidence interval; ref- reference category

**Table 4:** Final model 1 & 2- multivariate models of factors associated with oral SABA prescription at primary care asthma visits.

Parameter	Category	Final model 1		Final model 2	
		OR (95% CI)	F test	OR (95% CI)	F test
Asthma exacerbation	No	—	F(1,515) = 3.2	—	F(1,515) = 3.2
	Yes	1.7(0.9–3.2)		1.7(0.9–3.2)	
Oral corticosteroids	Not given	—	F(1,515) = 1.6	—	F(1,515) = 20.4***
	Given	1.6(0.8–3.1)		5.8(2.7–12.5)	
Inhaled steroids	Not given	—	F(1,515) = 1.4	—	F(1,515) = 1.4
	Given	0.5(0.1–1.6)		0.5(0.1–1.6)	
Inhaled SABA	Not given	—	F(1,515) = 22.0***	11.4(4.1–31.7)	F(1,515) = 22.0***
	Given	0.1(0.0–0.2)		—	
Respiratory tract infection	No	—	F(1,515) = 4.9*	—	F(1,515) = 4.9*
	Yes	2.2(1.1–4.3)		2.2(1.1–4.3)	
Sector	Public	—	F(1,515) = 0.2	—	F(1,515) = 18.6***
	Private	1.2(0.5–3.3)		6.5(2.8–15.3)	
Oral corticosteroids x inhaled SABA	Given oral corticosteroids x Given inhaled SABA	3.7(1.4–9.8)	F(1,515) = 11.1*	—	F(1,515) = 7.0*
	Given oral corticosteroids x Not given inhaled SABA	—		0.3(0.1–0.7)	
Inhaled SABA x sector	Given inhaled SABA x Private	5.2(1.7–15.8)	F(1,515) = 8.7*	—	-—F(1,515) = 8.7*
	Not given inhaled SABA x Private	—		0.2(0.1–0.6)	
Intercept		0.4(0.2–1.0)	F(1,515) = 3.5	0.0(0.0–0.1)	F(1,515) = 58.3*

Results based on multiple imputed datasets

*p<0.05

***p<0.001 based on logistic regression results

OR- odds ratio; CI- confidence interval; ref- reference category

The bivariate analyses in Table 3 presented evidence of a curvilinear relationship between age and oral SABA usage, where the usage was highest among patients with age between 20 and <40 years. The odds of receiving oral SABA was significantly higher among those who received oral corticosteroids (OR = 4.7, p<0.001). On the contrary, the likelihood of oral SABA being prescribed among those who received inhaled SABA was lower compared to those who did not receive the inhaled medication (OR = 0.2, p<0.001). A similar finding was also found among those receiving inhaled corticosteroids (OR = 0.09, p<0.001). Table 4 shows the results from the final models. Patient encounters with concomitant respiratory infections had significantly increased odds of being prescribed oral SABA compared to encounters without respiratory infections (OR = 2.2,p<0.05). The likelihood of receiving oral SABA was also higher among encounters with asthma exacerbation than those without exacerbation, although this association was not statistically significant at the 5% level (OR = 1.7, p = 0.07). The odds of receiving oral SABA were higher among those who also received oral corticosteroids compared to those did not receive oral corticosteroids; this finding was statistically significant among encounters with concomitant inhaled SABA prescription (OR: 5.8, p<0.001) but not statistically significant among those without concomitant inhaled SABA prescription (OR: 1.6, p = 0.2). The models also show that the odds of oral SABA prescription were higher in the private sector compared to the public sector; this finding was statistically significant among encounters with concomitant inhaled SABA prescription (OR: 6.5, p<0.001) but not among those without concomitant inhaled SABA prescription (OR:1.2, p = 0.4).

## Discussion

About 32.6 per 100 asthma encounters in primary care prescribed oral SABA for asthma management. Its use was found to be higher among encounters with concomitant respiratory infections, with concomitant prescription of oral corticosteroids and in the private sector.

Oral SABA use appeared as common as use of oral corticosteroids. Although its use was less common than inhaled SABA (50.2 per 100 asthma encounters), oral SABA use exceeded that of inhaled corticosteroids (23.4 per 100 asthma encounters), a main therapeutic agent for disease control. Such finding is consistent with other studies that also reported underutilisation of inhalers [28–30]. The underuse of inhaled corticosteroids (ICS) is an important concern as over 40% of adults and children with asthma in Malaysia have persistent symptoms [31, 32] which denote poor disease control and hence, the need for inhaled corticosteroids to reduce asthma symptoms, improve lung function [33], decrease airway hyperresponsiveness [34], controlling airway inflammation [35] and reducing asthma mortality [36]. In addition, early initiation and regular daily low dose ICS is highly effective in reducing asthma symptoms and asthma related exacerbations, hospitalisation and death [1]. The use of oral SABA in this non-resource poor country was found to be higher than a study conducted in Vietnam, a developing country, where 56.5% of the medication prescribed for physician diagnosed asthma was oral medication of which 33.7% were oral SABA (33.7%) [37]. This is not a practice encouraged as inhaled SABA and ICS are readily available in the country. Furthermore, oral SABA has a slower onset of action than inhaled SABA and a higher risk of side-effects [7–9], and its use should be kept to a minimum and discouraged. Instead, ICS, should be initiated early in disease control and inhaled SABA for symptomatic relief as necessary.

In the multivariate analysis, the prescription of oral corticosteroids was found to be a positive predictor of oral SABA prescription. This may suggest the notion of such combined treatment in asthma exacerbation. The association between these two prescriptions could also reflect the usage of oral dosage forms as a substitute for the inhaled form of the respective medications. There are several possible reasons why oral forms are preferred over the inhaled forms. First, the ease of prescription and use of oral medications may be deemed more appealing and feasible by both prescribers and users [16]. Compared to oral medications, patients need to be educated on the use of an inhaler where proper technique is also needed for drug delivery. This may pose a technical difficulty to some patients. Second, asthma is considered a social stigma and the usage of inhalers, especially the preventive inhalers, is seen as a declaration of having asthma for the rest of the patient’s life [38]. Third, inhaled medications, whether brand-name or generic, are more costly than the oral form [39–41]; this could pose as a barrier in patients who received treatment in the private sector where payment mode is mainly out of pocket. The presence of respiratory infection was also found to be a predictor of oral SABA usage in asthma. This is not surprising as respiratory infection is a precipitant of asthma exacerbation [42]. This reason is supported by the finding of patient visits with asthma exacerbation whose odds of being prescribed oral SABA were 70% higher compared to those without exacerbation, although these results were not statistically significant. Such prescribing practice of oral SABA is not supported by clinical evidence nor advocated by asthma guidelines.

We also found that, given the same patient characteristics, the likelihood of oral SABA prescription was higher in the private sector compared to the public sector. It is known that organisational factors influence clinical decision-making of health care providers [43, 44] and the systematic differences found between the public and private health systems in terms of governance, regulations and financing could attribute to the difference in the prescribing habits of the health care providers. Under the training system for doctors in Malaysia, all doctors are required to undergo compulsory internship and training at public hospitals before deciding to cross sector to the private sector or continue their service in the public sector. While the initial clinical practices may be similar, their practices would be transformed over time by the contrasting ecosystems that these individuals are exposed to within the respective health sectors. The governance and regulations of the public clinics are managed centrally and the clinical practices generally follow centrally-developed standard operating procedures along with standard monitoring of quality. On the contrary, the private sector consists of numerous competing, privately owned clinics, whose providers are accountable to investors and consumers with very few external accountability obligations to regulatory authorities and professional bodies. Private providers are neither integrated nor coordinated, and are regulated by the Private Healthcare Services and Facilities Act [45]. Although legislation is in place that requires all practicing doctors whether public or private sector, to be registered under the Medical Act 1971 [45], there is no accreditation process for quality assurance and standard maintenance in the provision of medical care. There is no regulation or requirement for doctors to fulfil certain number of hours of continual medical education and standard performance measurement system. The payment mechanism also differs between the two sectors: the heavily subsidised health care in the public sector allows patients to receive health care at almost no cost whereas the cost of health care in the private sector is mostly borne by patients via out of pocket expenditures. Therefore, cost becomes an important consideration that determines disease management and prescribing patterns of health care providers. The lack of quality assurance programs in the private sector, along with the consideration of cost and patient preference in asthma treatment, may have compelled the higher usage of oral SABA in the private sector compared to the public sector.

As a non-resource poor country, inhaled salbutamol and ICS are accessible and affordable to all in Malaysia; this is in line with one of the WHO Sustainable Development Goals (SDG) to nations for sustainable development [46]. In a survey of medicine prices availability and affordability in 2005 by Babar et al, inhaled salbutamol was found available in 80% of the public facilities [47]. On the other hand, among the private dispensing doctors, only up to 45% of them had inhaled salbutamol available in their practices. However, patients treated in the private sector have the option of purchasing inhalers from private retail pharmacies, where the lowest price generic equivalent (LPG) inhaled salbutamol was available in 96.9% of the retails [47]. Treatment affordability issue is non-existent in the public sector as medicines are provided free. In the private sector, a 100 microgram salbutamol generic inhaler cost just over half a day’s wages sold at retail pharmacies; this was calculated based on the daily wage of the lowest paid unskilled Malaysian government worker [18]. As for inhaled corticosteroids, a 200 microgram budesonide generic inhaler and a 100 microgram beclomethasone innovator inhaler sold at private pharmacies cost about a day’s wages and three days’ wages, respectively [18]. As barrier to receiving inhaled therapy is minimal, the prescription and use of inhaled medications early in the treatment should be widely encouraged. The recent suggestion to introduce a government health insurance scheme to address financial catastrophe amongst users of private health care may further improve accessibility of inhaled medicines for asthma as the scheme alleviates the financial burden of patients [48]. However, mere accessibility to inhaled SABA and ICS is not sufficient to promote its usage; other intervention steps are required as well. The National Essential Medicines List (NEML) [49], a national reference likened to the WHO EML which includes medicines that satisfy the health care needs of the population and hence should be made available at all times, should be reviewed for possible removal of oral SABA from the list; this is in sync with the deletion of the oral dosage forms from the WHO EML in 2011 [11]. With the review of NEML, the need for production, procurement, distribution and utilisation of oral SABA in the country would be reviewed. The health services also need to be organised to support long-term management of asthma; this includes capacity building and training of health providers to prescribe inhaled preventers and relievers instead of the oral alternatives and to provide effective patient education on the disease as well as use of inhalers [38].

Our study is limited by the cross-sectional nature; hence, the findings may differ when measured at different time frames. We were also unable to assess if the use of oral SABA for any given patient was a single or regular treatment for asthma nor do we have information on the indication and duration of treatment. This added information could reflect the appropriateness of the use of oral SABA as a mainstay in asthma management. Another limitation of the study is the lack of data on asthma control, which is an important determinant in prescribing. Our study also did not include data on medications dispensed from community pharmacies where patients may obtain medication supply. Nevertheless, the study has provided an important insight into the prescribing practices in asthma and possible inferences on the quality of asthma care. Future research would benefit from examining longitudinal data of asthma patients, where asthma treatment and outcomes are examined to assess disease control and impact.

## Conclusion

The use of oral SABA in asthma management in primary care in a non- resource poor setting is found to be common and was associated with undesirable clinical practices against a background of incoordination within a fragmented health system. Effective allocation of resources and transformation of the current clinical practices and health service delivery to promote the use of inhaled medications especially ICS are necessary and the use of oral SABA be discouraged.

## Supporting information

S1 Dataset Full, imputed dataset. (XLS)
